# Dr. Paris

**Published:** 1857-02

**Authors:** 


*Dr. Paris.*

We are sure our readers will be interested by the perusal of the following biographical sketch of Dr. Paris, copied from the *American Medical Monthly:*—

'Among the distinguished dead, for whom our profession has been called to mourn during the last month, is Dr. John A. Paris, late President of the College of Physicians, London, the following brief account of whose life we take from a London paper:—

"It is with very sincere regret that we announce the death of this excellent and distinguished man. Few men have run so long, and at the same time so honorable a career. For half a century precisely, Dr. Paris had practiced as a physician, and had risen to the very highest honors which it was in the power of his professional brethren to bestow. He was born at Cambridge on August 7, in the year 1785, and at twenty-two years of age he was elected Physician to the Westminster Hospital—a most distinguished honor for so young a man; and he continued in the active exercise of his professional duties until within a fortnight of his death. For fifty years, then—a considerable period even in the history of a nation—was the gentleman, to whose memory we would fain offer a slight tribute of respect, actually engaged in the alleviation of suffering and in the relief of afflicted humanity.

"To Dr. Paris, as is well known in the profession and to all persons more intimately acquainted with his career, the office of the physician was no hireling's work to be hurried through for the accumulation of a fortune or earning distinction. It was the business and glory of his life. When but fourteen years of age, he commenced his studies for the arduous profession on which he was about to enter, and followed them up with a zeal incredible in so young a person. When he had attained the ripe age of threescore years and ten, the old man, true to the resolution of the boy, voluntarily took upon himself the arduous duties of President of the Medical Council of the Board of Health, and with his own hand wrote the introductory report on the cholera of 1854.

"His personal history may be dismissed in a few brief sentences. Born at Cambridge, as we have said, in 1785, he became a member of Caius College, in the University, and graduated when very young in medicine. Among his contemporaries he was distinguished for the extent and elegance of his classical attainments. The Classical Tripos was not then in existence, and so, independently of the exigencies of his medical degree, he had not at the University any opportunity for displaying that fine and intimate knowledge of the writings of antiquity for which he was afterwards so distinguished both in private life and as President of the College of Physicians. From Cambridge he went to Edinburgh, then remarkable as a school of medicine, and was the friend and intimate companion of the many celebrated men who, in the first years of the century, had congregated at the Scottish capital. On his return to London, at the age of twenty-two, he was elected, as we before said, physician at the Westminster Hospital, but soon after vacated the appointment, as it was his wish to establish himself in the town of Penzance, in Cornwall.

"During his residence at Penzance, Dr. Paris distinguished himself as the founder of the Royal Geological Society of Cornwall; this, we believe, was the first Geological Society in England. When at Penzance, too, he gave to the miners the great boon of the *tamping-bar*, the instrument by which they are enabled to pursue their business amid inflammable gases, without the fear of striking fire from the rock. By this simple but admirable invention, Paris no doubt saved more lives than many heroes have destroyed. In the year 1810 he returned to London, and here, for forty-five or forty-six years he was actively occupied as a practicing physician. He was elected President of the College of Physicians in 1844, and this office he held until the hour of his death.

"Dr. Paris was not only known as a physician of the highest eminence—he was as remarkable for his literary ability. The *Life of Sir Humphrey Davy* will ever remain one of the classical biographies of the English language. In connection with Mr. Fonblanque he also wrote the *Medical Jurisprudence*, which has remained a text-book with lawyers until our own day. His works of a more professional character, were, his Treatise *On Diet*, which first brought him into notice, and which was published at a very early age; his *Pharmacologia*, which has run through more editions than most books; and his work on medical chemistry. Besides these and many other publications, his *Philosophy in Sport* has attained an enormous popularity, and with his life the motive for an *incognito*, which was never really maintained, has altogether terminated. In so brief a notice as the one to which we are necessarily limited by considerations of space, we can say but little more.

"The last ten days of Dr. Paris' life were spent in the midst of excruciating sufferings, which were borne with the most remarkable fortitude. His chief concern appeared to be to console and comfort those around him, who could ill disguise their grief at the impending and irreparable loss. His intellect remained to the last as clear as at any time of his life, and while power of speech remained nobody who listened to him could believe that the end was so near at hand. The public and the medical profession have suffered great loss in the death of John Ayrton Paris, one of the most disinterested, honorable, and able men who have ever practiced the profession of medicine. The grief of his own family and of those whom he honored with his friendship, is not matter of public concern, save in so far as it may serve to show how this wise and good man was honored and beloved by those who knew him best." '

